# A case report of eruptive pruritic papular porokeratosis with atopic dermatitis treated with upadacitinib: a novel therapeutic perspective

**DOI:** 10.3389/fmed.2025.1651033

**Published:** 2025-09-01

**Authors:** Lingyu Hu, Yujuan Xiao, Chenxi Zhao, Fanglu Lou

**Affiliations:** ^1^Chengdu University of Traditional Chinese Medicine Affiliated Hospital, Chengdu University of Traditional Chinese Medicine Clinical College, Chengdu University of Traditional Chinese Medicine, Chengdu, China; ^2^Department of Dermatology, The First Affiliated Hospital of Chongqing College of Traditional Chinese Medicine, Chongqing, China; ^3^Department of Dermatology, Chongqing Hospital of Traditional Chinese Medicine, Chongqing, China

**Keywords:** upadacitinib, eruptive pruritic papular porokeratosis, Janus kinase inhibitors, atopic dermatitis, porokeratosis

## Abstract

Eruptive pruritic papular porokeratosis is a rare subtype of porokeratosis, characterized by the appearance of intensely pruritic erythematous papules. Conventional therapies—including antihistamines, corticosteroids, and retinoids—often yield limited clinical benefit. This case report describes a 73-year-old Chinese man diagnosed with eruptive pruritic papular porokeratosis coexisting with moderate-to-severe atopic dermatitis. The patient developed widespread erythematous papules involving the scalp, trunk, and extremities, accompanied by severe pruritus and exudation secondary to scratching. Despite prior treatment with tofacitinib, acitretin, tripterygium glycosides, and antihistamines (epinastine and olopatadine), the patient exhibited a suboptimal clinical response. Consequently, treatment with upadacitinib, a selective Janus kinase 1 inhibitor, was initiated. Within one week, pruritus markedly improved, and the papules began to flatten. After one month of therapy, pruritus had nearly resolved, and the skin lesions showed progressive regression. The patient continued on a reduced dose of upadacitinib, without evidence of relapse or treatment-related adverse events during follow-up. This case highlights the potential of upadacitinib as an effective and well-tolerated therapeutic option for eruptive pruritic papular porokeratosis with concurrent atopic dermatitis, particularly in refractory cases. Further studies are warranted to evaluate its long-term efficacy and safety.

## 1 Introduction

Porokeratosis (PK) encompasses a group of rare dermatological disorders, which may be either acquired or hereditary, with a pathogenesis that is only partially elucidated. Current research underscores the significance of genetic factors in its development (1). Notably, emerging evidence suggests that PK may also result from a “second-hit” mechanism, wherein environmental triggers—such as medications, trauma, or infections—interact with underlying genetic predispositions to initiate or exacerbate the disease process (2). Eruptive pruritic papular porokeratos is (EPPP), a distinct clinical subtype of PK, was initially characterized by Kanzaki et al. (3). Clinically, EPPP manifests as an acute outbreak of annular papules with well-defined, hyperkeratotic margins and is notably associated with severe pruritus. This condition is often linked to persistent itching and carries a risk of malignant transformation (3). Unlike classic PK, which typically develops insidiously and is often asymptomatic, EPPP presents acutely with severe pruritus, which significantly affects patients’ quality of life. Although the precise pathogenesis of PK is not yet fully understood, recent reviews, such as the one conducted by Kostopoulos-Kanitakis and Kanitakis (1), have identified genetic mutations that impact the mevalonate pathway. This suggests that porokeratosis may constitute a heterogeneous group of related disorders rather than a singular disease entity. Histopathologically, PK is characterized by the presence of the cornoid lamella, which is a vertical arrangement of parakeratotic corneocytes within an orthokeratotic stratum corneum. This hallmark feature is typically observed at the peripheral margins of the lesions (1). However, in EPPP, additional histopathological features such as spongiosis, interface dermatitis, and a dense perivascular lymphocytic infiltrate in the dermis may be observed, indicating a heightened inflammatory response that is not typically present in other PK (1). A skin biopsy can confirm the diagnosis by identifying these histological findings. Currently, there is no universally effective treatment for PK, and patients frequently endure persistent symptoms that significantly diminish their quality of life.

Janus kinase (JAK) inhibitors represent a novel class of small-molecule agents increasingly used in the management of autoimmune and inflammatory diseases. Upadacitinib, a selective oral JAK1 inhibitor, has been approved by the U. S. Food and Drug Administration (FDA) for the treatment of moderate-to-severe atopic dermatitis (AD) (4). Although there have been prior reports of EPPP treated with other JAK inhibitors, such as abrocitinib and tofacitinib (5, 6), to the best of our knowledge, this is the first documented case of EPPP successfully managed with upadacitinib—a highly selective JAK1 inhibitor with a distinct pharmacological profile. Compared with tofacitinib, a pan-JAK inhibitor, and abrocitinib, another selective JAK1 inhibitor, upadacitinib demonstrates superior JAK1 selectivity and a more favorable long-term safety profile (7). Clinical and regulatory evaluations have indicated that upadacitinib is associated with a lower incidence of laboratory abnormalities and may carry a reduced potential risk of carcinogenicity (8, 9). Moreover, upadacitinib has shown greater efficacy and a more rapid onset of action in the treatment of various inflammatory dermatoses, particularly moderate-to-severe AD (4). In our case, the patient had a coexisting history of moderate-to-severe AD, which may have contributed to the favorable therapeutic response. This dual clinical benefit underscores the potential of upadacitinib as an effective treatment option for complex pruritic dermatoses with overlapping immunopathogenic features.

## 2 Case presentation

**Figure 1:**
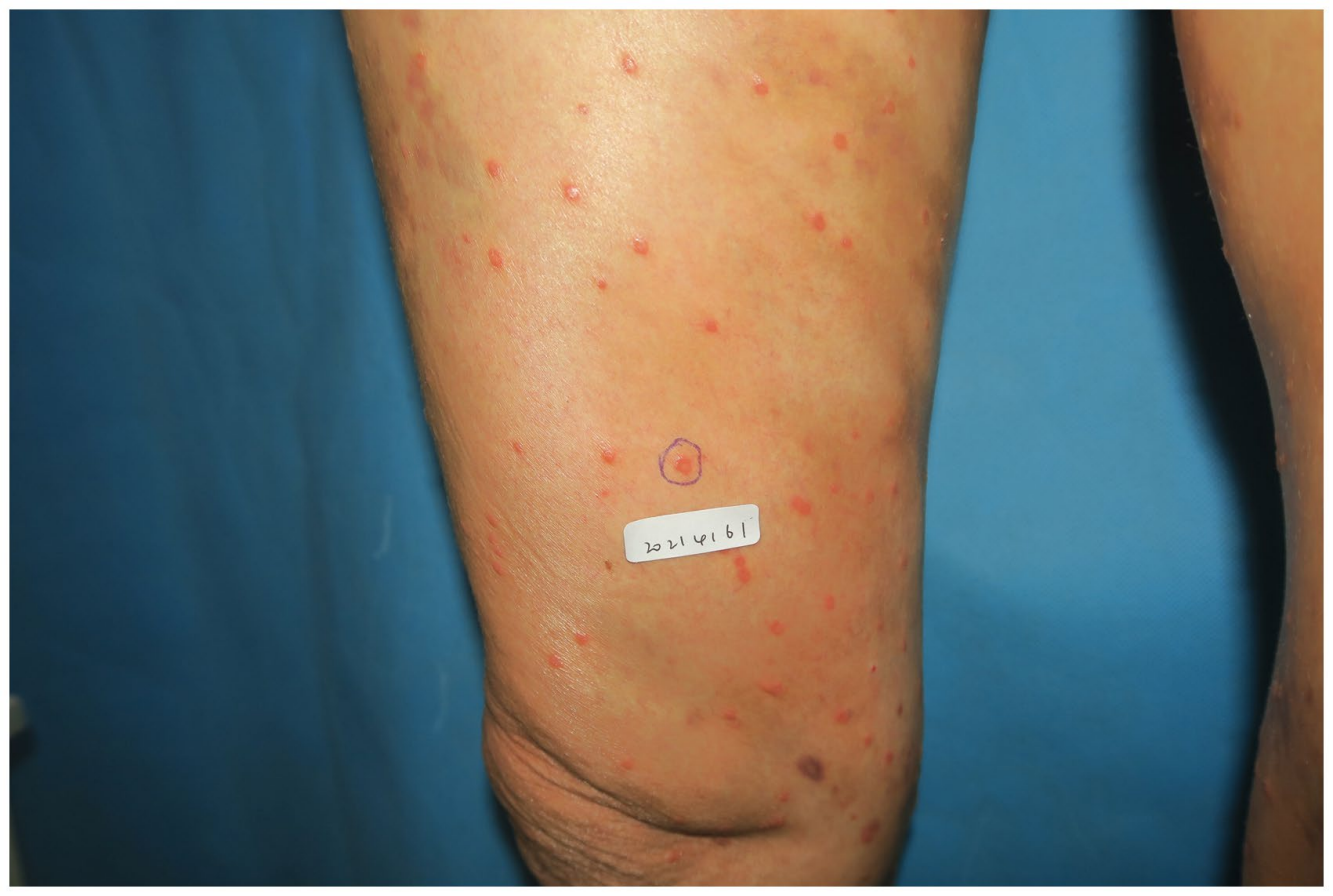
Numerous hyperkeratotic papules and erythematous plaques on the lower extremities, characterized by annular configurations with well-demarcated, hyperpigmented, and elevated borders (the circled area shows the biopsy site).

**Figure 2:**
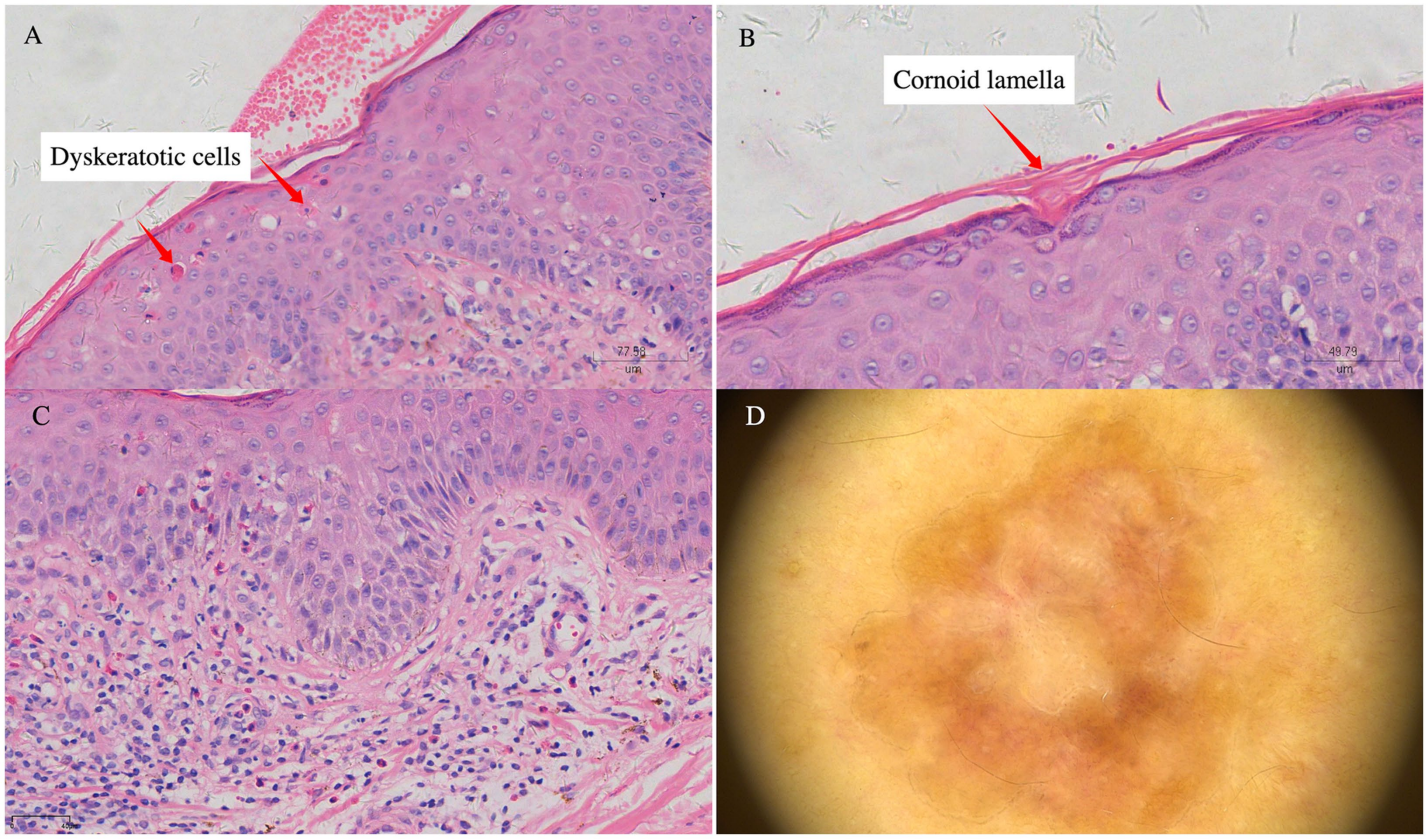
**(A)** Numerous dyskeratotic cells. **(B)** Cornoid lamella with parakeratosis. **(C)** Focal infiltration of lymphomononuclear cells and eosinophils in the superficial dermis and perivascular regions. **(D)** Dermoscopic image of the lesion on the right anterior chest of the patient: a well-demarcated lesion with central yellowish keratinous material and a peripheral hyperkeratotic rim.

A 73-year-old man with a 5-year history of grade 2 hypertension (maximum recorded blood pressure of 170 mmHg), managed with long-term oral nifedipine sustained-release therapy, and clinically stable chronic obstructive pulmonary disease (COPD), with no other hereditary disease history, presented with an acute onset of scattered erythematous macules and papules over the lower back and extremities (Figure 1), in the absence of any identifiable triggering factors. Some lesions exhibited slight scaling and were associated with severe pruritus. Localized areas showed mild serous exudation secondary to scratching. A skin biopsy and dermoscopic examination were performed at our institution (Figure 2), and based on the dermoscopic features, histopathological findings, and clinical presentation, a diagnosis of EPPP accompanied by eczema-like dermatitis was established (3).

**Figure 3:**
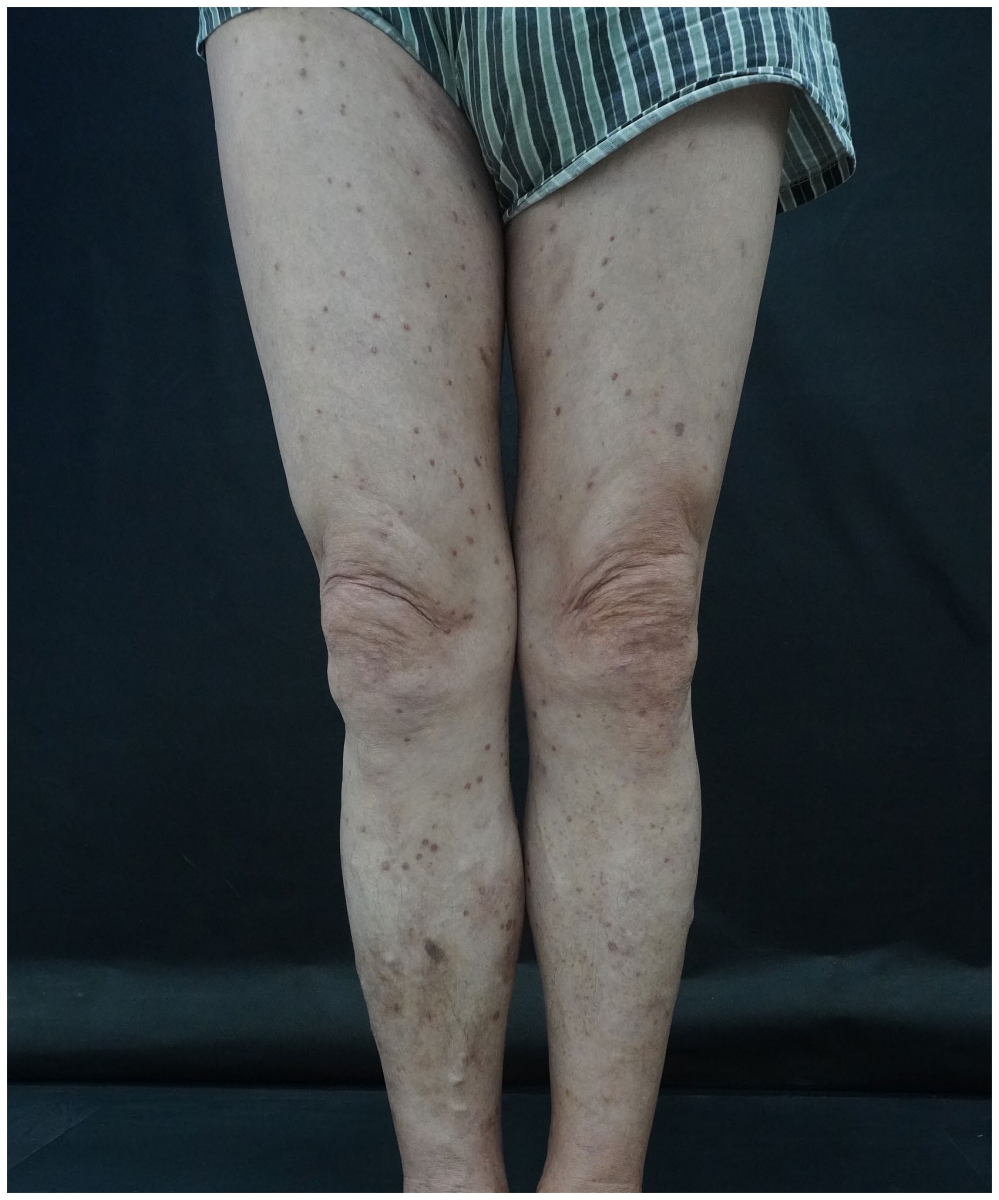
Numerous discrete to confluent erythematous papules and plaques distributed over the lower extremities, exhibiting annular morphology with raised, keratotic borders and adherent fine scales.

The patient was diagnosed with the condition in late 2021. Initial systemic therapy consisted of a combination regimen including: Tofacitinib citrate 5 mg orally, twice daily; Acitretin 25 mg orally, once daily; Tripterygium glycosides (a kind of Chinese herbal extract) 20 mg orally, three times daily; Epinastine 10 mg orally, once daily; Olopatadine 5 mg orally, twice daily. Despite two weeks of treatment, the patient showed no significant clinical improvement. The rash continued to spread, and pruritus worsened, indicating suboptimal therapeutic response. Throughout the disease, the patient experienced intermittent flare-ups. During each episode, the patient would receive the aforementioned medications at the outpatient clinic. Although these flare-ups were less severe than the initial one, they continued to cause significant discomfort. In June 2023, the patient returned with worsening symptoms. Dermatologic examination revealed erythematous macules (5–10 mm) and plaques or nodules (10–15 mm) on the scalp, trunk, and extremities (Figure 3). Some lesions had coalesced into larger confluent patches, and mild serous exudation was observed in areas affected by scratching. Pruritus remained intense and distressing. Given the rapid progression of the disease and its extensive systemic involvement, and based on both the patient’s current clinical manifestations and medical history, a diagnosis of EPPP was once again established. Differential diagnoses at that time included acute eczema, drug eruption, and cutaneous T-cell lymphoma; however, the overall clinical picture remained most consistent with EPPP. Consequently, the patient was admitted to the hospital for further diagnostic evaluation and therapeutic management.

After a renewed inquiry into the patient’s medical history, a history of allergic rhinitis was identified. Given the patient’s SCORAD score of 32.5, elevated eosinophil count (10.6%), a supplementary diagnosis of moderate-to-severe AD was established following Zhang’s Criteria for the Diagnosis of Atopic Dermatitis in China (10). Considering that EPPP has been reported in association with malignancies and infectious diseases, a comprehensive evaluation—including laboratory and imaging studies—was performed to rule out these conditions, with no abnormalities detected. In line with the 2020 Chinese Expert Consensus and American Academy of Dermatology guidelines, systemic therapies were initiated, including immunomodulatory and anti-inflammatory agents (tripterygium glycosides, acitretin), antihistamines (epinastine, olopatadine), corticosteroids (compound betamethasone), and compound glycyrrhizin, which possesses glucocorticoid-like and immunosuppressive effects (11, 12). These agents were administered concurrently during the first week of treatment. However, due to poor clinical response after one week, treatment was escalated to include upadacitinib, a selective JAK1 inhibitor approved for moderate-to-severe AD, particularly in cases refractory to conventional therapy. This decision aligns with the 2022 Chinese Guidelines for the Diagnosis and Treatment of Atopic Dermatitis, which recommend JAK inhibitors such as upadacitinib for patients who do not respond adequately to systemic immunosuppressants or corticosteroids (13). Moreover, large-scale clinical trials have confirmed the efficacy and safety of upadacitinib in treating moderate-to-severe AD in both adults and adolescents (4).

Following informed consent from the patient and family, extended-release upadacitinib (15 mg once daily) was initiated. Within one day of treatment, the patient reported noticeable relief of pruritus and flattening of papules and plaques. After one week, the pruritus had markedly improved, and the lesions showed significant regression. The patient’s condition steadily improved, and he was subsequently discharged. Pre-discharge laboratory tests revealed a total IgE level of 12.58 IU/mL and an eosinophil percentage of 2.8%.

**Figure 4:**
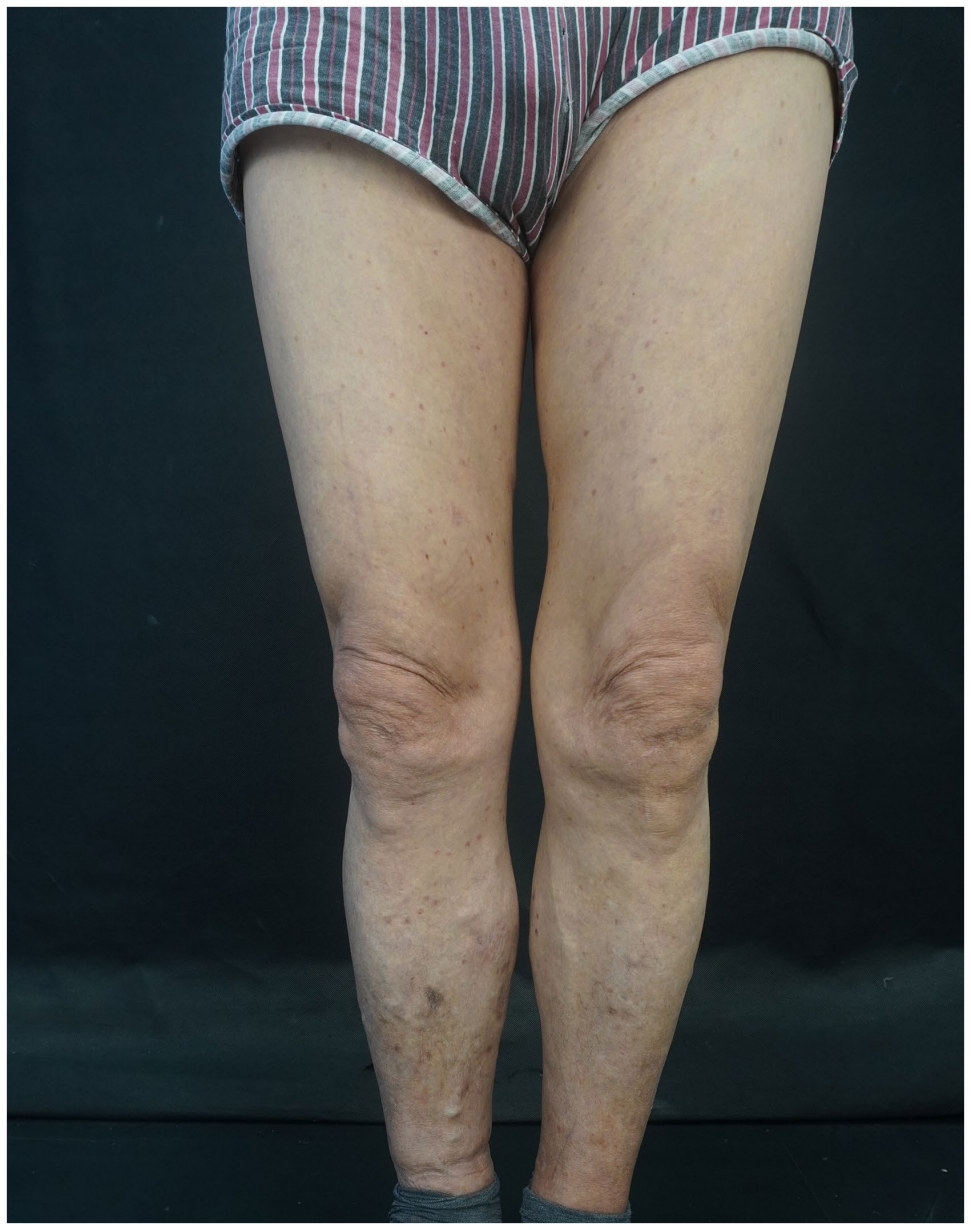
The photo was taken in June 2024, showing scattered dark red macules and areas of hyperpigmentation present on both lower extremities, with previous papular lesions having flattened.

Post-discharge, the patient was managed with a trial of upadacitinib (15 mg once daily) as monotherapy, which was attempted with gradual dose tapering and regular outpatient follow-up. Laboratory monitoring—including complete blood count, hepatic and renal function tests, myocardial enzymes, and D-dimer—revealed no treatment-related adverse events. Over a follow-up period exceeding one year, the disease remained well-controlled without relapse (Figure 4).

## 3 Discussion

In 2021, the patient underwent a pathological examination at Chongqing Traditional Chinese Medicine Hospital and was diagnosed with EPPP, based on histopathological findings and the clinical presentation of intense pruritus. Despite treatment with various anti-inflammatory and antihistamine agents, the therapeutic effect was suboptimal. In 2023, the patient returned to our hospital due to persistent symptoms. After one week of conventional therapy with no improvement, treatment with upadacitinib was initiated. The disease was brought under control, and the symptoms gradually improved. The patient has now been maintained on upadacitinib for over one year, with no signs of relapse or adverse drug reactions. Based on this case, we propose the following considerations:

### 3.1 JAK1 inhibition as a potential strategy for inflammatory keratinization disorders

EPPP is a rare, inflammatory variant of porokeratosis, typically affecting the elderly and characterized by a sudden onset of intensely pruritic papules. As described by Morgado-Carrasco et al. (14), EPPP may represent an inflammatory subtype of disseminated superficial porokeratosis (DSP). Compared to classic DSP, inflammatory EPPP is more responsive to systemic immunomodulatory therapies like JAK inhibitors.

In this case, the patient was diagnosed with both EPPP and moderate-to-severe AD. Upadacitinib, a selective JAK1 inhibitor, effectively suppresses Th2 cytokines such as IL-4, IL-13, and IL-31. It was initiated after failure of multiple other therapies, including tofacitinib. The patient experienced rapid relief from severe pruritus and marked improvement in both eczematous and hyperkeratotic lesions. While the precise pathogenesis of EPPP remains unclear, this favorable response highlights the potential role of JAK1-targeted therapy in modulating inflammatory skin conditions beyond AD. From a therapeutic standpoint, upadacitinib not only alleviated pruritus but also improved the keratinizing lesions. This dual effect suggests that selective JAK1 inhibition may offer therapeutic potential in inflammatory keratinization disorders such as EPPP.

### 3.2 Understanding the divergent efficacy of Upadacitinib and Tofacitinib

JAK1 inhibition by upadacitinib may disrupt pathological crosstalk between keratinocytes and immune cells, thereby ameliorating inflammatory lesions and hyperkeratotic margins. Emerging evidence implicates cytokines such as thymic stromal lymphopoietin (TSLP) and interleukin-31 (IL-31)—both of which signal through the JAK1 pathway—in exacerbating keratinocyte dysfunction and pruritus (14–17). The JAK/STAT signaling axis is central to immune regulation, transmitting extracellular cytokine signals to the nucleus to modulate gene expression. In particular, TSLP, an epithelial cell-derived cytokine, is known to activate dendritic cells and promote Th2 polarization, while IL-31 is directly involved in the induction of pruritus and skin barrier disruption. Both cytokines exert their biological effects through JAK1-dependent signaling (16). Thus, selective inhibition of JAK1 by upadacitinib may alleviate pruritus and restore keratinocyte homeostasis by interrupting these pro-inflammatory cytokine pathways.

Notably, the patient in this case did not respond to tofacitinib, a pan-JAK inhibitor that targets JAK1, JAK3, and to a lesser extent JAK2 (6). In contrast, significant improvement was observed following treatment with upadacitinib, a highly selective JAK1 inhibitor. This differential efficacy may be due to the more focused inhibition of JAK1-specific pathways by upadacitinib, resulting in more efficient suppression of pruritogenic and keratinocyte-activating cytokines such as IL-4, IL-13, IL-31, and TSLP (16). Although prior reports have shown that tofacitinib can be effective in some cases of EPPP (6), our findings suggest that selective JAK1 inhibition may offer a more targeted and potentially superior therapeutic strategy in certain patients with inflammatory keratinization disorders.

### 3.3 Therapeutic advantages of upadacitinib

Topical corticosteroids and oral antihistamines are commonly used to treat PK/EPPP; however, most cases respond poorly to these conventional therapies (18). In this case, upadacitinib effectively alleviated pruritus and improved skin lesions simultaneously. This targeted approach achieved clinical benefit without the need for multiple medications, thereby minimizing the risks associated with polypharmacy. Although the patient was elderly and had underlying disease, no adverse events—such as hepatotoxicity or thrombosis—were observed during treatment. This outcome aligns with the favorable safety profile reported in clinical trials of upadacitinib for AD (4).

In addition to conventional therapies, several other treatment options for porokeratosis have been explored, including topical 5-fluorouracil, imiquimod, retinoids, cryotherapy, and laser therapy, though their efficacy is often limited or inconsistent (19). Recently, topical lovastatin-cholesterol has emerged as a promising treatment, particularly for porokeratosis subtypes associated with mutations in the mevalonate pathway (20). This therapy targets the underlying metabolic dysfunction by restoring cholesterol and inhibiting toxic intermediate accumulation (1, 20).

### 3.4 Limitations and future directions

Although this case provides preliminary evidence supporting the efficacy of upadacitinib in EPPP, several limitations should be acknowledged. As a single-case report, the findings lack generalizability and should be interpreted with caution. Moreover, mechanistic insights remain limited. Future investigations—including single-cell RNA sequencing of EPPP lesions—are warranted to elucidate the direct effects of JAK1 inhibition on keratinocyte differentiation, proliferation, and immune interactions.

## 4 Conclusion

This report highlights upadacitinib as a promising and fast-acting therapeutic option for EPPP, particularly in patients with Th2-driven conditions such as AD. Future research should prioritize controlled clinical trials to confirm its efficacy and safety, as well as translational studies to uncover the interactions between the JAK1 signaling pathway and the pathogenesis of keratinizing disorders, especially in patients with EPPP/PK.

## Data Availability

The raw data supporting the conclusions of this article will be made available by the authors, without undue reservation.
